# A Wearable Morphic Sensor for Unobtrusive Heart Rate and Respiratory Rate Monitoring in ICU Patients: A Pilot Validation Study

**DOI:** 10.3390/s26196052

**Published:** 2026-09-24

**Authors:** Titus Jayarathna, Caitlin Polley, Paul P. Breen, Gaetano D. Gargiulo, Anders Aneman

**Affiliations:** 1 The MARCS Institute for Brain, Behaviour and Development, Western Sydney University, Locked Bag 1797, Penrith, NSW 2751, Australia; 2 School of Engineering, Western Sydney University, Locked Bag 1797, Penrith, NSW 2751, Australia; c.polley@westernsydney.edu.au (C.P.); g.gargiulo@westernsydney.edu.au (G.D.G.); 3 Department of Intensive Care, Liverpool Hospital, Elizabeth Street, Liverpool, NSW 2170, Australia; anders.aneman@health.nsw.gov.au

**Keywords:** wearable electronics, morphic sensors, forcecardiographic signal, discrete wavelet transform, ICU monitoring, vital signs

## Abstract

Purpose: This pilot study evaluated the real-world accuracy of a novel wearable morphic sensor system for unobtrusive, continuous heart rate (HR) and respiratory rate (RR) monitoring in critically ill patients within an intensive care unit (ICU). Methods: A hybrid piezoresistive/piezoelectric disk-shaped sensor was placed on patients’ backs to capture cardiorespiratory activity via forcecardiographic principles. Data were acquired from 15 ICU patients using a custom wireless recorder. The piezoelectric channel was processed using discrete wavelet transform and an adaptive peak detection algorithm. Derived HR and RR were compared against reference measurements from clinical patient monitors and a ventilator. Results: Bland–Altman analysis showed a mean bias of −0.39 ± 1.99 beats per minute for HR and 0.06 ± 0.90 breaths per minute for RR. Linear regression revealed strong correlation with clinical monitors (HR R-squared = 0.982; RR R-squared = 0.966). The device’s RR performance matched the agreement between two independent clinical reference devices. Conclusion: The proposed system met the pre-specified bias and standard deviation criteria for HR and RR monitoring in bed-bound ICU patients. Its skin-adhesive-free design enables unobtrusive, continuous vital sign surveillance without direct skin contact. These findings support ICU monitoring. Step-down ward and ambulatory applications remain to be validated in future studies.

## 1. Introduction

Vital sign measurement is a consistent and critical component of modern healthcare. From general practice to intensive care, it is normal and expected that various physiological metrics will be measured and considered, impacting treatment plans. The quality and frequency of these measurements relate in some part to healthcare outcomes for patients. It is notable, however, that as patients transition from one healthcare setting to another, the frequency of measurement changes. For example, consider a patient within intensive care, where monitoring is continuous, transferring to a general ward setting where single observations may be made on a 4 hourly basis. This represents a significant drop in monitoring frequency, but it must be considered in the context of the ward environment. Cost is also a consideration: intensive care monitoring equipment is expensive. Furthermore, these monitors are typically tethered to the patient, limiting ambulation and increasing fall risk if the patient leaves the bed. Ideally, the quality of physiological monitoring would be equal across all clinical settings and implemented in a way that allows free movement.

In an effort to maintain more continuous monitoring in the ward and other settings, several wearable devices have been developed, and others have come to market. The majority of available solutions require skin contact via electrodes for electrocardiogram or impedance measurement or via optical means for photoplethysmography [1,2]. Contact between skin and sensor is not always reliable, and over extended periods can have deleterious skin responses [3]. Other mechanisms have been explored to extract physiological data in a contactless manner via camera or radar-based systems [4,5]. In contrast, invasive implantable sensors take their measurements directly from the organ of interest [6]. The use of inertial motion units to extract similar metrics through the capture of body movement, e.g., ballistocardiogram, has also been widely examined [7,8,9]. This type of technology is low-cost to implement and sits in a middle ground of being neither contactless nor requiring direct skin contact. Respiratory impedance plethysmography (RIP) bands, often used in sleep studies [10,11], may be considered within the same category. Such technologies may generally be regarded as morphic sensors, whereby the work of the body, e.g., cardiac activity or respiration, changes the morphology of the body and this morphic change is mechanically translated to a sensor. A significant advantage of such systems is that direct physical contact between the sensor and skin is not required.

This work fits within the morphic sensor category and explores the potential of an alternative sensing mechanism to extract physiological metrics. Morphic sensors, including those based on fabric deformation and soft material strain, offer promising avenues for wearable monitoring due to their conformal, unobtrusive nature and their ability to sense both respiratory and cardiac activity through mechanical coupling [12,13]. Table 1 summarizes recent studies on non-contact cardiorespiratory monitoring, comparing sensing technologies, experimental protocols, and key performance metrics.

Our proposed cardio-respiratory Dot sensor has the form of a 30 mm radius (9 mm thickness) disk. This device is a sensor fusion of a piezoresistive element and a piezoelectric element that we previously employed in separate studies [20]. The combined structure is capable of detecting mechanical cardiac events, including aortic valve opening and pre-ejection period, through forcecardiographic principles [21]. The sensor is designed to be placed at the back of the patient such that the mechanical deformation travel via the soft silicone medium to the sensor. However, for this study, given the patients are in ICU, the sensor was placed inside a sterile ultrasound cover and contact with bare skin was avoided altogether by shielding the sensor with a thick, clean hospital towel. The sensor was placed approximately in the apical position (by a medical practitioner) at the back of the patient. No active control of mechanical preload was applied. The FSR channel recorded loading but was not used to adjust placement. There are several reasons why such a system may be useful and preferable to alternatives. The device can be easily attached and detached and is wearable over light clothing, eliminating adhesion requirements of other solutions. Unlike wrist- or finger-worn devices, the chest or back mounted position allows unencumbered access to the hands, wrists, and arms, which may be required for the insertion of cannulas or needle-less access connectors.

In this pilot study, we aimed to determine whether our morphic sensor system is sufficiently accurate for respiratory rate (RR) and heart rate (HR) monitoring in critically ill patients within an ICU environment. Given the distinct physiological challenges in ICU patients compared to healthy individuals, we sought to validate the system’s accuracy against established clinical devices. We hypothesized that the morphic sensor system would achieve a mean bias within ±2 beats per minute (bpm) for HR and ±2 breaths per minute (brpm) for RR, with a standard deviation of differences no greater than 2 bpm for HR and 2 brpm for RR, compared with clinical reference devices. These targets align with clinical standards for ICU monitoring, such as AAMI EC13 for HR [22] and available commercial device specifications [23], and are supported by studies reporting ±2–3 bpm for HR and ±1–3 brpm for RR as clinically acceptable [24,25]. The 95% limits of agreement were not required to fall within ±2 bpm/brpm, the pre-specified criterion applied to bias and the standard deviation of differences.

The novelty of this work lies in three aspects. First, we present a hybrid piezoresistive/piezoelectric sensor that operates without direct skin contact, eliminating adhesive-related skin issues and allowing unobtrusive monitoring. Second, we validate this sensor in a real-world ICU setting with critically ill patients, unlike many prior studies that use healthy volunteers or mannequins. Third, we demonstrate that a single-channel mechanically coupled signal can reliably extract both heart rate and respiratory rate using a lightweight wavelet-based adaptive peak detection pipeline, achieving accuracy comparable to the agreement between two independent clinical gold standards.

## 2. Methods

### 2.1. Device Design and Implementation

The wireless data recording unit, shown in Figure 1d, was manufactured on a single PCB containing the application processor (CC2640R2F, Texas Instruments, Dallas, TX, USA), a 24-bit analog-to-digital converter (ADS1247, Texas Instruments, Dallas, TX, USA), and additional power management integrated circuits (chargers and regulators). The data recorder connects to the Dot sensor via a wired connection, and the recording unit connects to an Android app via Bluetooth to stream data for quick validation. The simulated AC response of the instrumentation circuit is shown in Figure 2.

### 2.2. Data Acquisition and Pre-Processing

The sensor was evaluated on 15 patients admitted to the Liverpool Hospital Intensive Care Unit (ICU), NSW, Australia (Ethics approval: South Western Sydney Local Health District, reference ETH00124). Physiological signals were recorded and stored as raw data in binary format on a microSD card on our custom recording device (Figure 1d). The device sampled three channels at 180 Hz: the piezoelectric sensor, a force-sensitive resistor (FSR), and a third auxiliary channel. Only the piezoelectric channel, which carried the strongest cardiorespiratory signal, was used for further analysis. The FSR is used to characterize the pressure applied to the sensor, and to measure the respiration with low-frequency pressure changes. The third auxiliary channel was sampled but not used in this study; it is reserved as a separate input for future applications requiring a synchronized analog channel. The 180 Hz sampling rate was selected because it provides approximately sixfold oversampling of the highest frequency of interest (30 Hz) and comfortably satisfies the Nyquist requirement for the target frequency range.

The system clock was derived from the internal oscillator of the CC2640R2F microcontroller, and MCU clock interrupts were used to resample the ADC output to the target rate. Clock accuracy was characterized on the same recording platform by comparing the observed sample count against an external function generator reference (KEYSIGHT 33210A, Keysight Technologies, Santa Rosa, CA, USA) over a 5-day 18-h continuous acquisition. The effective sampling rate was 399.9416 Hz for a nominal 400 Hz setting, corresponding to a relative timing error of 0.0146%. Over the same acquisition, the missed-sample rate was 0.00065% (325 missed samples out of 49,962,800 expected). For an 8 h recording, the worst-case cumulative clock drift is approximately 4.2 s. This is small relative to the alignment offset search used in post-processing and does not affect breath-to-breath or beat-to-beat rate calculations, which use local windows.

A total of 15 datasets were collected across multiple sessions. One dataset was excluded due to data corruption during acquisition (SD card failure), and another due to extremely low signal-to-noise ratio (SNR) caused by the sensor being placed too loosely against the body, resulting in 13 valid recordings. No numerical SNR or signal-quality threshold was pre-specified; the low-SNR dataset was excluded after acquisition based on visual inspection, in which the signal appeared as a flat line containing only noise. This sample size is typical for pilot feasibility studies in ICU monitoring settings published recently [26,27].

Heart Rate (HR) and Respiratory Rate (RR) were simultaneously recorded using clinical patient monitors (Philips IntelliVue MP70 and MX750, Philips Medizin Systeme Böblingen GmbH, Böblingen, Germany) and a ventilator (the Hamilton-C6, Hamilton Medical AG, Bonaduz, Switzerland). At 15-min intervals, a timestamped video recording of both clinical devices was taken (Figure 1b); this was repeated at least five times for each patient. The Dot device and the videos were synchronized in post-processing analysis. The patient monitor recorded both HR and RR and was used as the primary clinical reference for HR and RR. The ventilator provided an independent RR measurement and was used as a secondary clinical comparator for RR, allowing the agreement between two clinical devices to be contextualised.

Raw data were imported into MATLAB R2023a (MathWorks, Natick, MA, USA) via a custom interface for further processing. Initial signal inspection showed a low-frequency respiratory component with superimposed sharper features attributable to cardiac activity. Both HR and RR were extracted from this single-channel waveform using a shared adaptive peak detection framework.

### 2.3. Signal Decomposition via Discrete Wavelet Transform

The single-channel waveform was decomposed using a Discrete Wavelet Transform (DWT) with Daubechies-4 (db4) wavelets, a standard choice for physiological signal denoising [28,29,30]. Decomposition used the symmetric extension mode (the MATLAB wavedec default), which mirrors the signal at each boundary to limit edge artifacts. No explicit detrending or demeaning was applied before decomposition; low-frequency drift was attenuated by the instrumentation high-pass response (0.13 Hz cutoff, Figure 2) and by the wavelet approximation itself.

For HR extraction, detail coefficients at levels 4 and 5 were reconstructed and summed to emphasize cardiac mechanical activity. At the acquisition rate of $F_{s}=180$ Hz, these bands correspond to approximately 5.6–11.3 Hz (D4) and 2.8–5.6 Hz (D5); the D4 + D5 reconstruction therefore retains approximately 2.8–11.3 Hz. For RR extraction, approximation coefficients at level 6 were retained, isolating approximately 0–1.4 Hz, which contains the respiratory band of interest (typically 0.1–0.7 Hz).

To enhance the respiratory signal without over-smoothing physiologically relevant events, a moving average filter (1.5 s window) was applied, followed by a second-order Butterworth high-pass filter (0.1 Hz cutoff, zero-phase filtfilt) to remove residual baseline drift. An adaptive smoothing rule then selectively replaced portions of the smoothed signal with the corresponding level-6 approximation values: where the absolute difference between the raw approximation and its moving average exceeded mean+4SD, the smoothed samples over the preceding 1.5 s window were replaced by the original approximation, preserving transient features such as sharp breaths or motion artifacts.

No global amplitude normalization was applied. All detection thresholds were instead computed relative to the local peak-to-peak amplitude within each processing block (Section 2.4). Amplitude outliers were suppressed by percentile trimming before feature extraction: 1st–99th percentile for the HR derivative-squared computation, and 10th–90th percentile within each RR block. Block-edge handling and the peak detection parameters are described in Section 2.4.

### 2.4. Adaptive Peak Detection Algorithm

Heart and respiration rates were estimated using a shared adaptive peak detection algorithm, as outlined in Algorithm 1. The method was applied to non-overlapping 60-s blocks that were processed independently; blocks were neither overlapped nor cross-smoothed during detection. Peaks from all blocks were concatenated and sorted only after detection, and rates were then recomputed globally. HR derivative-squared smoothing used movmean with ‘Endpoints’,‘shrink’ to avoid edge distortion at block boundaries. Because rates are recomputed globally after concatenation, at most one peak per block edge can be affected.
**Algorithm 1** Adaptive Peak Detection for HR or RR Estimation.**Require:** 1-min signal segment x(t), sampling rate $F_{s}$ **Ensure:** Peak times, instantaneous and smoothed rate (HR or RR)
  1:Remove amplitude outliers using percentile trimming (1st–99th percentile for HR; 10th–90th percentile for RR).  2:Compute a moving peak-to-peak amplitude over a sliding window to estimate signal dynamics.  3:Set initial peak detection threshold as one-quarter of the mean peak-to-peak amplitude.  4:Set ${\mathrm{threshold}}_{\min}\leftarrow 0.10\times {\mathrm{threshold}}_{\mathrm{initial}}$.  5:Define starting maximum peak distance from the maximum expected physiological rate (120 bpm for HR; 40 brpm for RR), and set maxDist←startDist.  6:Set $\mathrm{minDist}\leftarrow F_{s}\times 60/180$ (HR) or $F_{s}\times 60/60$ (RR).  7:**for** up to 10 iterations **do**  8:      Detect peaks using current threshold and maximum peak distance.  9:      **if** number of detected peaks <3 (HR) or <2 (RR) **then**10:           Relax threshold and maximum distance; **continue**.11:      **end if**12:      Compute instantaneous rate: $r_{i}=60/\left(t_{i+1}-t_{i}\right)$.13:      Apply median filtering (window size = 5) to obtain smoothed rate $\tilde{r}$.14:      Compute mismatch: $|\mathrm{mean}\left(r\right)-\mathrm{mean}\left(\tilde{r}\right)|$.15:      **if** mismatch ≤ 2 bpm/brpm **then**16:           Accept results; **break**.17:      **else if** $\mathrm{mean}\left(r\right)<\mathrm{mean}\left(\tilde{r}\right)$ **then**18:           $\mathrm{threshold}\leftarrow \max(0.80\times \mathrm{threshold},\;{\mathrm{threshold}}_{\min})$19:           maxDist←max(0.90×maxDist,minDist)20:      **else**21:           maxDist←min(maxDist/0.90,startDist)22:           threshold←threshold/0.8023:      **end if**24:      **if** $\mathrm{threshold}={\mathrm{threshold}}_{\min}$ **and** maxDist=minDist **then**25:           **break**26:      **end if**27:**end for**28:**return** Final peak indices, times, instantaneous rate, and smoothed rate.


Key features of this method include iterative adjustment of detection parameters and a convergence criterion based on the mean–median mismatch of instantaneous rate estimates. The rationale is that missed peaks produce longer inter-peak intervals, lowering the estimated rate, while false positives create shorter intervals, raising the estimated rate. By comparing the raw mean rate (sensitive to both extremes) to the median-smoothed rate (robust to outliers), the detection threshold and maximum peak distance are adjusted iteratively, with convergence indicating reliable detection [31,32].

At each iteration, the detection threshold and the maximum peak distance are updated multiplicatively: when the mean rate falls below the median-smoothed rate (indicating missed peaks), the threshold is reduced by a factor of 0.80 and the maximum peak distance by a factor of 0.90, both bounded below by predefined physiological limits. When the mean rate exceeds the median-smoothed rate (indicating false positives), the maximum peak distance is increased by dividing by 0.90 (up to the starting value) and the threshold is increased by dividing by 0.80. The starting threshold was set to one-quarter of the mean peak-to-peak amplitude within each block, and the starting maximum peak distance was set from the maximum expected physiological rate (120 bpm for HR, 40 brpm for RR). Iteration terminates when the mean–median mismatch is ≤2 bpm/brpm, when a hard parameter limit is reached, or after 10 iterations, whichever occurs first.

The parameter values reported above were fixed prior to the evaluation of the present dataset and were not tuned on it. The algorithm was developed and its parameter ranges established on separate pilot recordings and on the physiological bounds reported in the literature [31,32]; the same fixed parameters were then applied to all 13 datasets reported here. No per-patient or per-dataset parameter adjustment was performed, and no held-out tuning set was used. Development, parameter selection, and performance evaluation were therefore kept separate, though a formal train/test split was not possible given the pilot sample size.

While adaptive detection has been previously applied to wearable ECG [33] and PPG [34] signals, those implementations incorporate only partial aspects of the present approach. The full algorithm described here, combining iterative parameter tuning with a mean–median convergence check, represents a novel contribution aimed at improving robustness under variable signal quality.

### 2.5. Postprocessing and Smoothing

Once peaks were identified across all 1 min segments, global peak times were concatenated and sorted. Instantaneous rate (in bpm or brpm) was computed using the inverse of inter-peak intervals and further smoothed using a moving average filter (window length = 10 samples). Both raw and smoothed rate traces were retained for downstream analysis.

### 2.6. Bland–Altman Analysis

Bland–Altman analysis was performed to evaluate the agreement between estimated and reference HR and RR measurements. Bias was defined as the mean difference (estimate minus reference) and the 95% limits of agreement as bias ± 1.96 standard deviation (SD). Because multiple measurements were obtained from each patient, these observations are not independent. Confidence intervals for bias, limits of agreement, MAE, ARMS, and regression parameters were therefore obtained by cluster bootstrap, resampling patients with replacement (5000 iterations). As a sensitivity analysis, per-patient mean differences (n = 13) were also computed to assess between-patient consistency. Data from 13 datasets (excluding 2 as described above) were concatenated for the primary analysis.

### 2.7. Linear Regression Analysis

To evaluate the performance of RR estimation, linear regression analysis was conducted using aggregated data from 13 recording sessions. Estimated RR values were regressed against reference RR measurements obtained from clinical-grade devices to assess their linear agreement. Additionally, a secondary regression analysis was conducted between the ventilator and patient monitor-derived respiration rates. This analysis aimed to quantify the level of agreement between these independent clinical sources. For both comparisons, the slope and intercept of the linear fit were used to characterize proportional and constant bias, while the coefficient of determination ($R^{2}$) quantified the strength of linear correlation. Mean Absolute Error (MAE) was also calculated to capture average absolute deviations. Together, these analyses allowed assessment of the estimation model’s fidelity relative to ground truth, while also contextualizing its performance within the variability present between established clinical measurement systems.

## 3. Results

### 3.1. Signal Processing Pipeline

Figure 3 shows the data processing steps and their effect on the signal and peak detection performance for heart rate detection, and Figure 4 shows the data processing steps for respiratory rate detection.

For HR, the raw piezoelectric waveform was decomposed via DWT, and the D4 + D5 components (approximately 3–11 Hz) were combined to isolate cardiac activity. The derivative-squared signal was smoothed to enhance periodic peaks, yielding a clean cardiac profile separable by dominant peaks. For RR, the level-6 DWT approximation was extracted to isolate low-frequency respiration, followed by moving-average and adaptive filtering to obtain a smooth respiratory waveform. Detected HR and RR peaks are indicated in red and yellow, respectively.

### 3.2. Bland-Altman Analysis and Linear Regression

Figure 5 shows the Bland–Altman plots for HR and RR. The concatenated analysis showed an HR bias of −0.39 bpm (95% CI −0.91 to 0.06) with 95% limits of agreement of −4.30 to 3.52 bpm (lower limit 95% CI −6.46 to −1.80; upper limit 95% CI 1.90 to 4.77). RR bias was 0.06 brpm (95% CI −0.17 to 0.30) with 95% limits of agreement of −1.72 to 1.83 brpm (lower limit 95% CI −2.18 to −1.25; upper limit 95% CI 1.27 to 2.39). The mean absolute error (MAE) was 1.28 bpm for HR (95% CI 0.73 to 2.01) and 0.66 brpm for RR (95% CI 0.49 to 0.88). The average root mean square error (ARMS) was 2.02 bpm for HR (95% CI 0.95 to 2.96) and 0.90 brpm for RR (95% CI 0.68 to 1.14).

Linear regression analysis is shown in Figure 6. Strong agreement was observed for HR (slope 0.976, 95% CI 0.924 to 1.002; intercept 1.674, 95% CI −0.312 to 5.707; $R^{2}=0.982$) and RR (slope 0.905, 95% CI 0.846 to 0.940; intercept 1.690, 95% CI 1.146 to 2.765; $R^{2}=0.966$). RR estimates outperformed the agreement between the ventilator and patient monitor, both considered clinical gold standards, which showed a lower $R^{2}$ of 0.745 and higher MAE of 2.051 brpm.

In the per-patient sensitivity analysis (n=13), the HR bias was −0.39 bpm with limits of agreement −2.24 to 1.47 bpm, and the RR bias was 0.05 brpm with limits of agreement −0.96 to 1.07 brpm. The near-identical bias and narrower per-patient limits of agreement indicate that between-patient variability is small and that most of the observed variability arises from within-patient differences across individual measurements.

## 4. Discussion

This pilot study presents a comprehensive evaluation of a novel wearable sensor platform designed for continuous heart rate (HR) and respiratory rate (RR) monitoring in critically ill ICU patients. Using recordings from 13 valid datasets collected in real clinical environments, the proposed signal processing pipeline combining discrete wavelet transform-based signal decomposition and an adaptive peak detection algorithm demonstrated strong agreement with established clinical reference devices.

The HR and RR estimates showed minimal bias and narrow limits of agreement compared to gold standard measurements from patient monitors, with mean errors below 1 bpm or brpm. Importantly, the device’s RR performance matched the agreement between two independent clinical gold standards (ventilator and patient monitor), suggesting that the proposed approach can match or improve upon the consistency of existing monitoring equipment.

These results support our initial hypothesis that the morphic sensor system would achieve clinically acceptable accuracy. The observed mean bias and standard deviation of differences met the pre-specified criterion of ±2 bpm for HR and ±2 brpm for RR (HR: bias −0.39 bpm, SD 1.99 bpm; RR: bias 0.06 brpm, SD 0.90 brpm). The finding that the device’s RR performance matched the agreement between two established clinical reference devices further supports the consistency of the proposed system, providing preliminary evidence for its core premise. The limits of agreement were narrow for RR (−1.72 to 1.83 brpm) and approximately twice as wide for HR (−4.30 to 3.52 bpm). This is expected, as cardiac mechanical signals are smaller in amplitude and more indirect than the large, slow mechanical deformation produced by respiration. The observed HR limits of agreement fall within the ±5 bpm benchmark commonly used for HR monitoring [35]. This is consistent with the relationship LoA=bias±1.96×SD. Under the pre-specified criterion of bias ≤2 and SD ≤2, the theoretical worst-case limits of agreement could be as wide as approximately ±5.92 bpm, so the ±5 bpm benchmark is stricter than the bias/SD criterion. Nevertheless, the observed HR limits of agreement met this stricter benchmark. Thus, the predefined ±2 criterion was met in terms of bias and standard deviation, not in terms of the 95% limits of agreement.

Data collection in a challenging ICU setting, involving sedated or non-responsive patients who could not provide feedback on sensor placement or comfort, highlights the robustness of the sensor system. Only two datasets were excluded: one due to data corruption and another due to poor sensor contact, which defines the practical lower coupling limit rather than a need for controlled preload. The pilot data demonstrated that the device is resilient to common practical issues encountered in bedside monitoring. Importantly, the lack of controlled preload is a strength of this design: the system operated without straps, adhesives, or controlled coupling, relying only on the patient’s body weight and the sensor’s placement between the back and the bed.

The adaptive peak detection method’s iterative parameter tuning, driven by a mean–median rate convergence criterion, effectively handled variations in signal morphology and noise, allowing accurate detection without relying on computationally intensive machine learning or frequency-domain techniques. This classical time-domain processing approach, enhanced by careful wavelet-based filtering, provides a computationally efficient and interpretable solution suitable for real-time clinical use.

Our results also confirm that a single-channel sensor, when combined with tailored signal processing, can extract both cardiac and respiratory signals reliably, simplifying hardware design while maintaining performance. The observed performance consistency across a broad range of clinically relevant HR (60–121 bpm) and RR (8–28 brpm) further supports the device’s applicability for continuous patient monitoring.

### 4.1. Validated Scope

The present results apply to bed-bound ICU patients with the sensor placed between the back and the bed. They do not establish accuracy during ambulation, in step-down wards, or in home monitoring. These settings remain future applications and require separate validation.

### 4.2. Novelty and Clinical Importance

The clinical importance of this work is that it provides a practical path to continuous, unobtrusive vital sign monitoring in bed-bound ICU patients. By removing the need for skin adhesives or tethered monitors, the system may reduce skin complications, improve patient comfort, and enable monitoring in settings where continuous surveillance is currently limited. The novelty lies in the sensor fusion design, the validated accuracy in real ICU conditions, and the signal processing pipeline that extracts both HR and RR from a single mechanical channel.

### 4.3. Limitations

Several limitations of our study should be noted. First, the HR and RR values from the ventilator and patient monitor were not recorded simultaneously, but rather sampled sequentially within approximately one minute of each other. As these devices report average rates, transient changes in physiology during this interval may have introduced discrepancies, particularly during rapid transitions in RR. This could partially explain the weaker agreement between the two clinical references compared to our device’s agreement with either one.

Furthermore, RR estimation algorithms differ in how they classify short inhalation bursts, such as whether gasping counts as a complete respiratory cycle. Since there is no standardization across devices in this regard, it is not unexpected that gold-standard instruments also show variability.

Another limitation arises from the ICU environment, where non-responsive patients could not provide feedback on sensor placement or comfort, potentially affecting signal quality. Two datasets were excluded—one due to data corruption from SD card failure and another due to low signal-to-noise ratio (SNR) caused by poor sensor contact, exacerbated by placement inside a sterile ultrasound cover with a towel for infection control. The exclusion of the low-SNR dataset was based on post hoc visual inspection rather than a pre-specified numerical threshold; future studies should define quantitative signal-quality criteria prospectively. These challenges highlight practical issues in ICU monitoring that can impact data quality. To mitigate these, future iterations could: (1) use the wireless feature for real-time signal visualization via the Android app to adjust sensor placement for optimal SNR; (2) employ industrial-grade microSD cards to prevent data corruption; (3) implement visual or auditory alerts on the recording unit to warn of low-quality data; and (4) incorporate redundant sensor arrays or backup recorders to enhance reliability.

## 5. Conclusions

In summary, this study validates the proposed sensor platform and signal processing pipeline as a promising solution for unobtrusive, continuous cardio-respiratory monitoring in ICU patients. Its accuracy, robustness, and efficiency support further clinical evaluation. Future work should explore longer-term monitoring, integration with hospital workflows, and expansion to other physiological parameters to further enhance patient care.

## Figures and Tables

**Figure 1:**
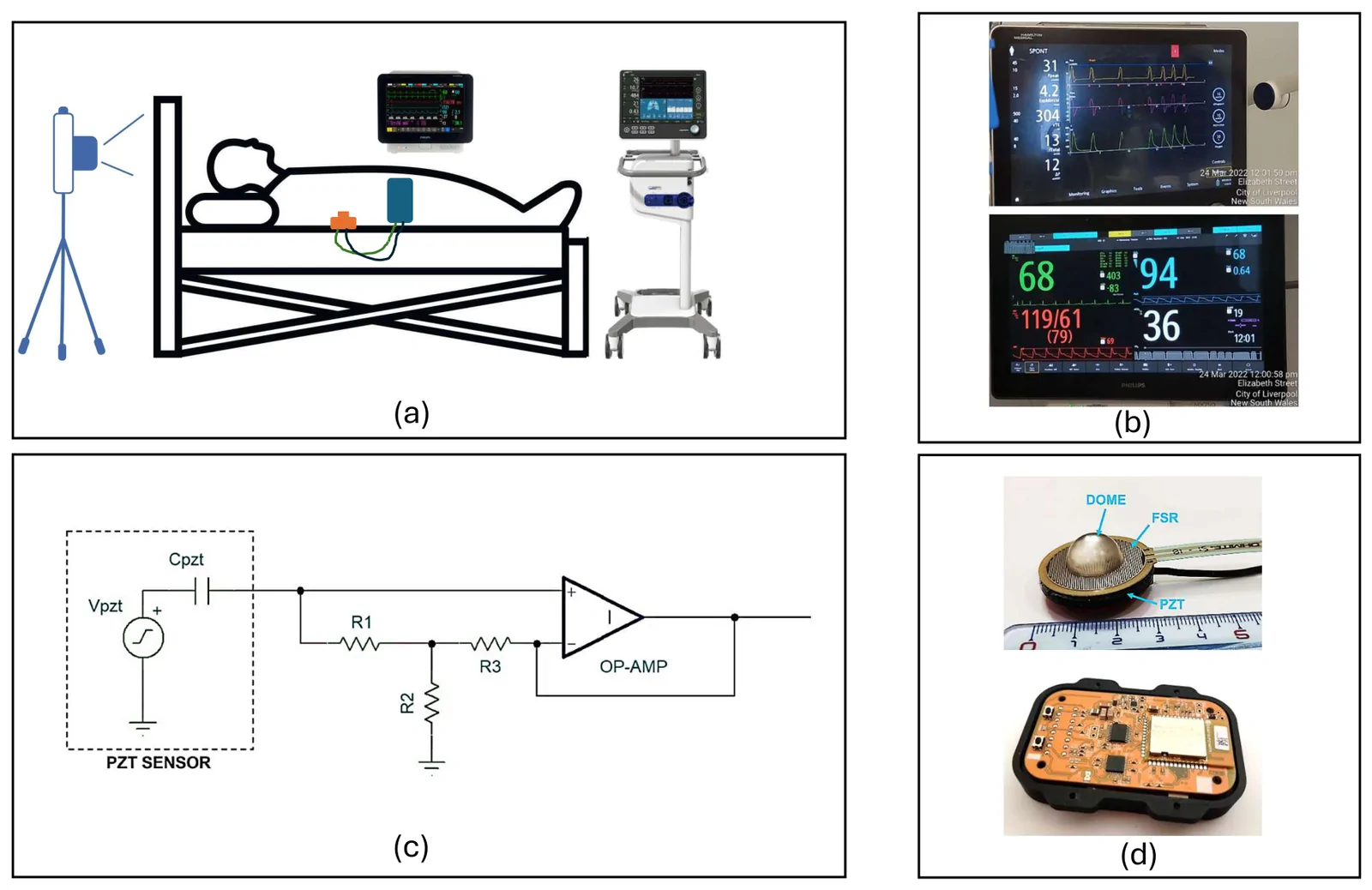
Device design and experiment setup. (**a**) The ICU bed setup with a patient monitor, ventilator, the recording camera, the sensor placed between the patient and the bed, and the attached wireless data recorder. (**b**) Example screenshots from recorded video of the ventilator and patient monitor. (**c**) Instrumentation circuit of the piezoelectric sensor. The piezoelectric element ($C_{pzt}=10$ nF) was AC-coupled to the non-inverting input of a unity-gain operational amplifier. A T-network ($R_{1}=10$ MΩ, $R_{2}=100$ kΩ, $R_{3}=100$ Ω) provided the DC bias path. The Op-Amp is TL081 (Texas Instruments, Dallas, TX, USA). (**d**) The Dot sensor, which contains a stacked force-sensitive resistor (FSR) and a piezoelectric sensor (PZT), together with the PCB of the wireless data recorder.

**Figure 2:**
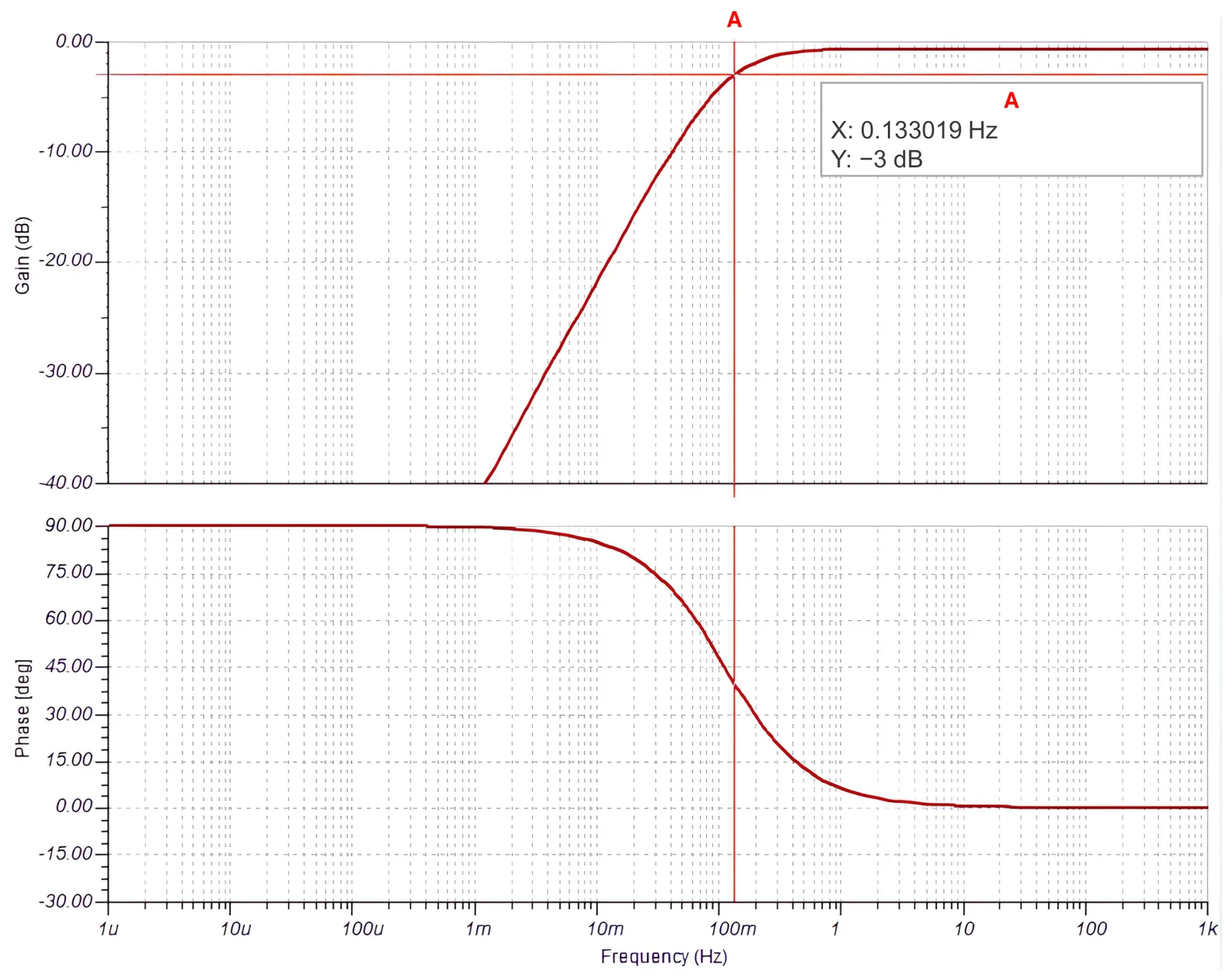
Simulated AC transfer function of the instrumentation circuit between 1 µHz and 1 kHz. The cutoff frequency is 0.13 Hz. The phase is $40.3^{\circ }$ at the cutoff frequency, approaches zero above 2 Hz, and remains constant up to 1 kHz.

**Figure 3:**
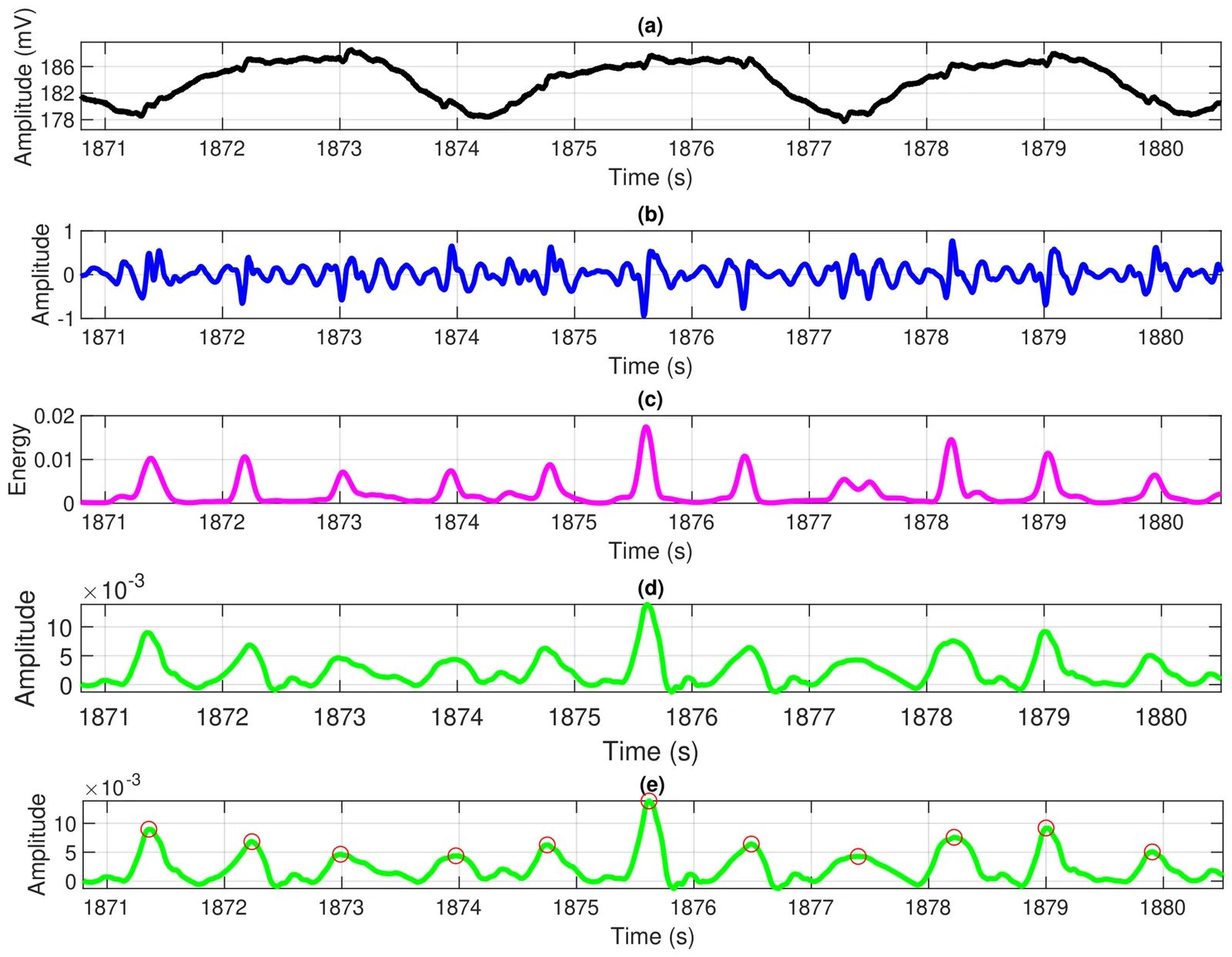
Heart rate signal pathway overview: (**a**) raw signal; (**b**) bandpass-filtered signal (D4 + D5, 3–11 Hz); (**c**) smoothed energy of signal (squared derivative); (**d**) reconstructed signal (DWT approximation); (**e**) final signal with detected heartbeat peaks circled in red.

**Figure 4:**
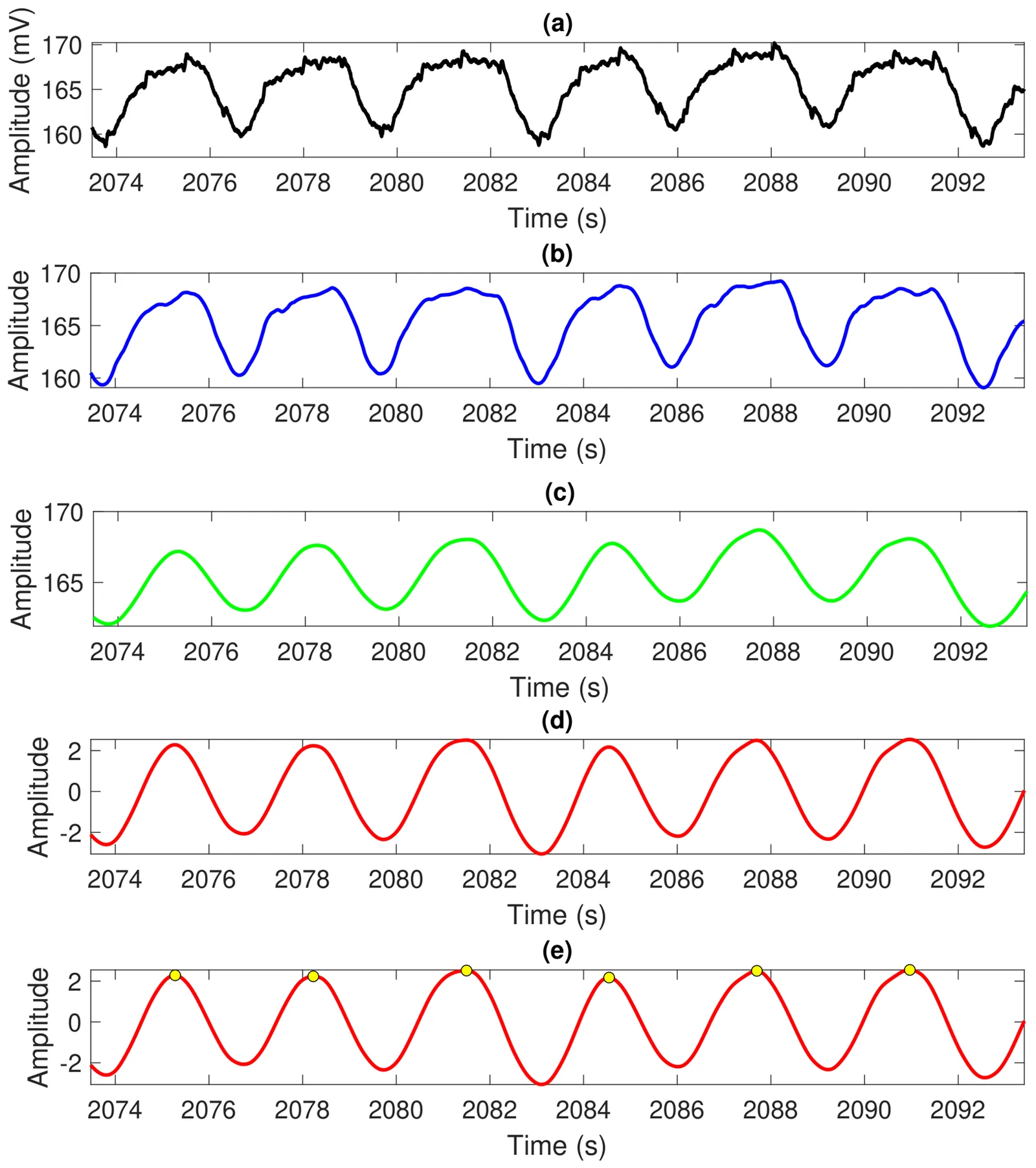
Respiratory rate signal pathway overview: (**a**) raw signal; (**b**) extracted respiration signal (DWT Level 6 Approximation); (**c**) moving average smoothed respiration signal; (**d**) adaptive filtered respiration signal; (**e**) final signal with detected respiration peaks circled in yellow.

**Figure 5:**
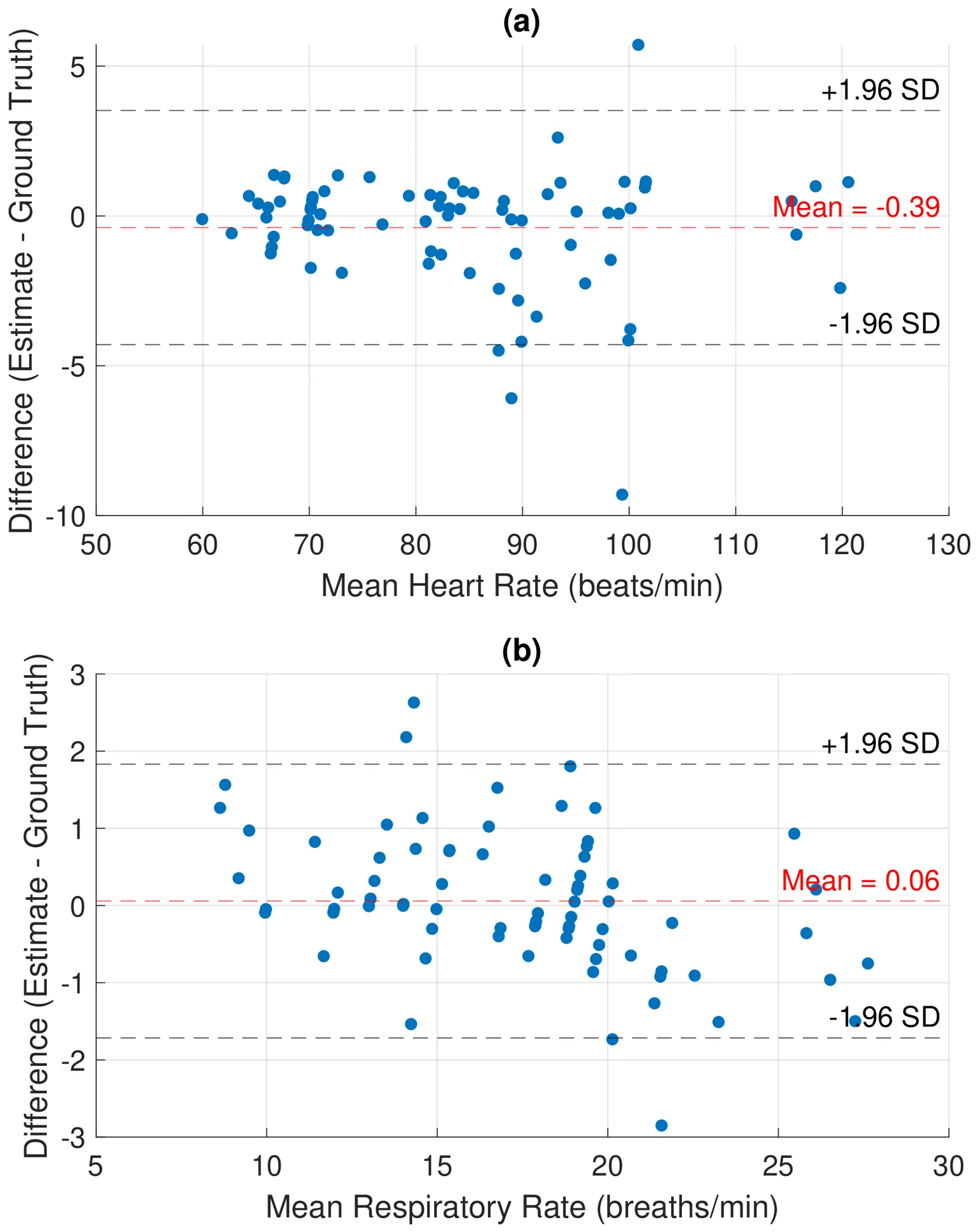
Bland–Altman analysis: (**a**) Bland–Altman plot of the HR results; (**b**) Bland–Altman plot of the RR results.

**Figure 6:**
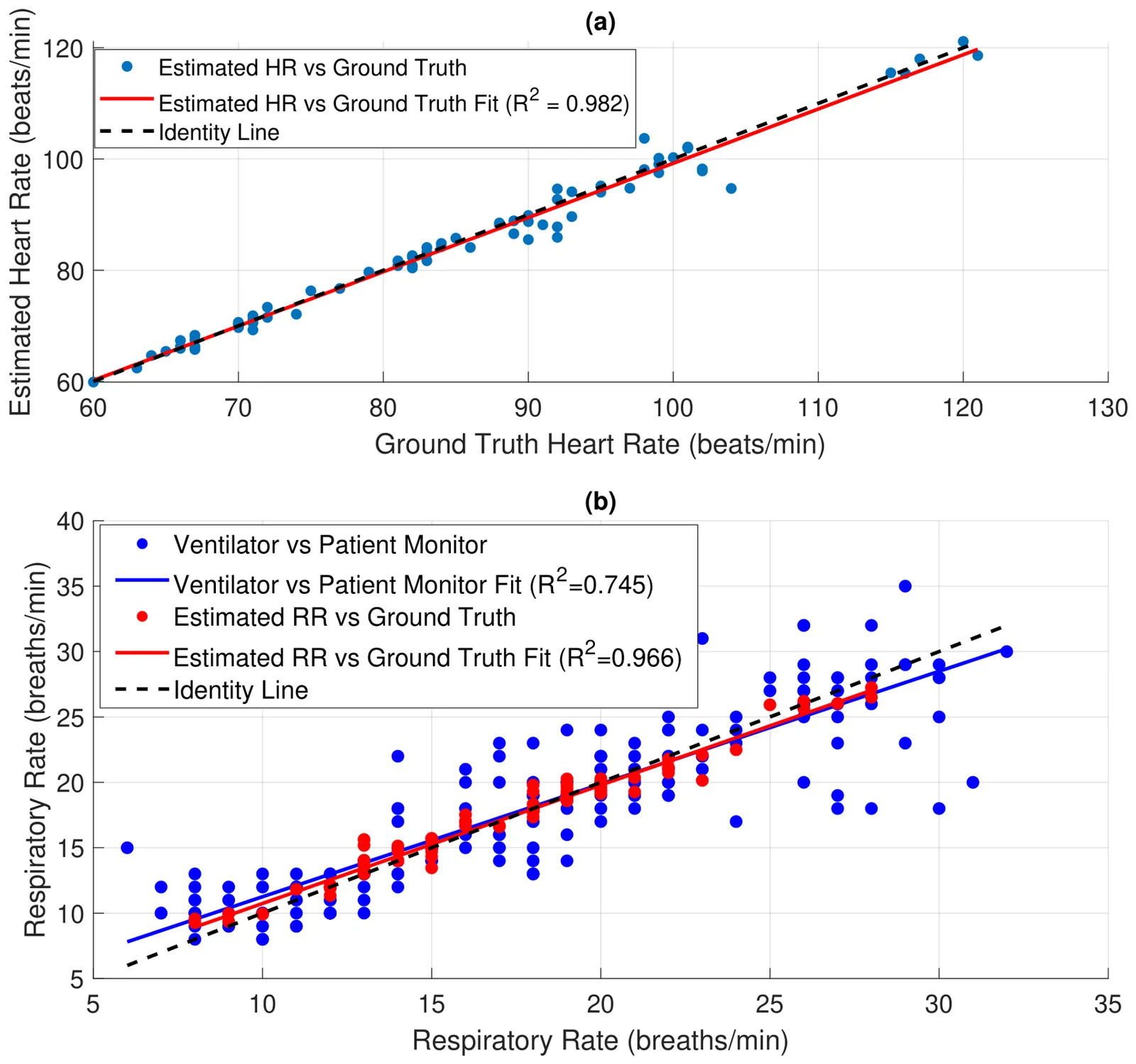
Linear regression analysis comparing HR and RR measurements across devices. (**a**) Scatter plot of HR values from the patient monitor (reference) versus those estimated by the Dot device. (**b**) Scatter plots of RR values comparing the patient monitor to the ventilator (blue, reference comparison) and the patient monitor to the Dot device (red).

**Table 1:** Selected studies on non-contact monitoring of heart rate and respiratory rate.

Study (Year)	Technology	Participants/Setting	Parameters	Reported Performance	Remarks
Wang et al. (2024) [14]	CCTV camera (Panasonic WV-SC384H, 18× zoom)	25 ICU patients	HR, RR	HR MAE = 1.3 bpm (94.5% success at ±2 bpm), RR MAE = 0.7 brpm (96.7% at ±3 brpm)	Complements contact monitoring during sensor removal; motion artifacts main error source; usage depends on ambient lighting level
Arasteh et al. (2023) [15]	UWB radar (Xethru X2M200)	55 children, PICU and sleep lab	HR, RR	HR MAE = 7.3 bpm (r = 0.77); RR MAE = 2.63 brpm (r = 0.81)	Works in total darkness; no contact; simultaneous HR/RR; moderate HR error and limited by distance
De Tommasi et al. (2023) [16]	Fiber Bragg grating-based instrumented mattress	8 healthy volunteers (lab setting)	HR	MAPE <1.1% (quiet breathing), <2.6% (tachypnea); LOA ≤3.6 bpm	No skin contact; limited to bed-bound use; requires subject on mattress
Cay et al. (2022) [17]	e-textile chest belt with pressure sensors and IMU	Simulated preterm infant (NICU mannequin) + 5 healthy adults	RR	RR MAE = 0.89 brpm	Not yet validated on actual neonates; limited to RR/apnea (no HR); wearable (not fully non-contact)
Villarroel et al. (2019) [18]	RGB video camera with multi-task CNN	30 preterm infants, NICU	HR, RR	HR MAE = 2.3 bpm (76% valid time); RR MAE = 3.5 brpm (82% valid time)	Fully non-contact; works under ambient light; requires visible skin; not validated in dark/night conditions
Jarchi et al. (2018) [19]	Wearable tri-axial accelerometer (chest/arm)	10 post-ICU patients, ward setting	RR	MAE = 2.6 brpm vs. PPG-based RR	Low-cost; only estimates RR (no HR); requires wearable sensor (not fully non-contact)
Our work (2026)	Hybrid piezoresistive/piezoelectric morphic sensor	13 ICU patients, real-world clinical setting	HR, RR	HR: bias = −0.39±1.99 bpm ($R^{2}=0.982$); RR: bias = 0.06±0.90 brpm ($R^{2}=0.966$)	Wearable, no skin contact required; validated against clinical gold standards in ICU; enables unobtrusive continuous monitoring

Notes: bpm: beats per minute; brpm: breaths per minute; NICU: Neonatal Intensive Care Unit; PICU: Pediatric Intensive Care Unit; CNN: Convolutional Neural Network; UWB: Ultra-Wideband; IMU: Inertial Measurement Unit; MAE: Mean Absolute Error; MAPE: Mean Absolute Percentage Error; LOA: Limits of Agreement; PPG: Photoplethysmography; SpO2: Peripheral capillary oxygen saturation.

## Data Availability

The datasets generated and analysed during the current study are available from the corresponding author on reasonable request.

## Abbreviations

The following abbreviations are used in this manuscript:

ICU	Intensive Care Unit
HR	Heart Rate
RR	Respiratory Rate
DWT	Discrete Wavelet Transform
bpm	beats per minute
brpm	breaths per minute
MAE	Mean Absolute Error
ARMS	Average Root Mean Square Error
